# Anaphylaxis After Consumption of Guar Gum-Containing Food: A Report of Two Cases

**DOI:** 10.7759/cureus.28022

**Published:** 2022-08-15

**Authors:** Betul Dumanoglu, Gulistan Alpagat, Merve Poyraz, Sumeyra Alan Yalim, Ayse Baccioglu

**Affiliations:** 1 Department of Immunology and Allergic Diseases, Kirikkale University Faculty of Medicine, Kirikkale, TUR

**Keywords:** epinephrine, anaphylaxis, allergy, guar gum, food additives

## Abstract

Guar gum is a food additive that acts as a thickening agent. Although the relationship between guar gum and occupational rhinitis/asthma has already been established, only very few cases of anaphylaxis were associated with guar gum ingestion. We present two examples of anaphylaxis induced by guar gum. Both cases were successfully treated with adrenaline. Moreover, serum-specific immunoglobulin E (IgE) with the culprit agent was detected in blood samples. To the best of our knowledge, here we present the first case of class 6 guar gum-specific IgE-positive anaphylaxis. This report aims to raise awareness of rare food additive allergies such as guar gum.

## Introduction

There are a wide variety of synthetic and natural agents used as food additives. They serve many technical purposes, such as preserving, flavoring, sweetening, thickening, coloring, and stabilizing foods; most cannot be consumed alone [1]. E numbers were assigned to food additives in the European Union to identify them, and labels must include names, functions, and E numbers of additives [2]. Gums act as stabilizing and thickening ingredients in foods, making mixtures more viscous without altering their flavor [3]. Foods may include a variety of different gums. Guar gum, carob gum, tragacanth, and Arabic gum are the most known gums. Guar gum (E 412) is derived from a plant (*Cyamopsis tetragonoloba*), which is a member of the Leguminosae family [1,3]. It is frequently used in the bakery industry to keep the bread from going stale and increase its shelf life and quality [4]. A report from the World Health Organization and the Joint FAO/WHO Expert Committee on Food Additives says there is no limit on gums' acceptable daily intake values (ADI, mg/kg). It also mentions that they don't have any toxicological effects on human health [5]. While it is well known that gums cause occupational asthma and rhinitis, they rarely cause allergic reactions after ingestion [1]. Here we present two cases of anaphylaxis after consumption of guar gum-containing food.

## Case presentation

Case 1

A 23-year-old woman without any known allergies ate two packages of Indomie Noodle® (Indonesia), mixing special flavor and vegetable flavor at 3:00 pm. Thirty minutes later, she started to itch, followed by hives. The first admission to the emergency room was at 6:30 pm (approximately three-and-half hours post-exposure), with the development of severe itching and generalized urticaria. Her complaints regressed after pheniramine maleate and methylprednisolone intravenous (IV) treatment. The next day at 6:00 am (15 hours post-exposure), she presented generalized urticaria, difficulty breathing, bronchospasm, dizziness, and syncope. She was transferred to the emergency department by ambulance with these symptoms and findings for the second time within 24 hours, and adrenaline administrated immediately. After the first shot of adrenaline, almost all symptoms resolved rapidly, except moderate urticaria. Therefore, she was referred to the allergy department at 11:30 am. At the time of the allergy evaluation, unilateral angioedema of the tongue was seen in addition to hives. Because of the persistence of her symptoms for a long time, protracted anaphylaxis was considered. She was admitted to the inpatient unit and monitored for four days. After completely resolving all symptoms, an epinephrine auto-injector was prescribed and discharged from the hospital.

Diagnostic tests

Tryptase level was measured as 30.6 ug/L (UniCAP®, Pharmacia, Uppsala, Sweden) when she was initially admitted to allergy department, while basal tryptase was 2.82 ug/L. During the second visit one month later, firstly, prick tests were performed with a standard aeroallergen panel and 12 food allergens (banana, cow milk, egg white, egg yolk, peanut, walnut, tomato, kiwi, sesame, hazelnut, wheat flour, soybean flour). They were all negative. 

Secondly, we questioned daily meals and investigated ingredients which may be included in the ingested noodle. Wheat flour is the main nutritious component of noodles and also contains many additives. During this period, she was able to ingest gluten, the primary allergen, as well as other additives besides guar gum, without experiencing any adverse reactions.

Then blood samples were taken for allergen-specific IgE: caraway, mace, cardamom, clove, basil, fennel seed, ginger, anise, egg white, milk, fish, wheat, peanut, and soybean specific IgEs were all negative; in contrast, guar gum was detected class 6 positive (114kuA/L). An oral provocation test was not performed since specific IgE for guar gum was shown in the blood. As far as we know, this is the first case report of guar gum hypersensitivity with such a high specific IgE value and protracted anaphylaxis.

Case 2

A 19-year-old male patient presented to the outpatient clinic complaining of swollen lips and shortness of breath 3-4 hours after consuming cornbread one week ago. He has perennial allergic rhinitis with worsening symptoms being around the birds at his home. He also suffered intermittent dyspnea for two months.

Diagnostic tests

First, he was evaluated for asthma and allergic rhinitis. The bronchodilator reversibility test was positive. Prick tests for grass mix (5*6 mm), mugwort (3*4 mm), and cat dander (4*4 mm) were all positive, and peripheral eosinophilia was detected (1022/mm3). Allergen-specific Ig E was found to be class 2 positive (0.7 - 3.5 kuA/L) for most pollens such as *lolium perenne*, *secale cereale*, artemisia vulgaris, and grass mix (*anthoxanthum odoratum*, *lolium perenne*, *phleum pretense*, *secale cereale*, *holcus lanatus*), but class 3 (3.5 - 17.5 kuA/L) positive for bird feathers mix (budgerigar feathers, canary feathers, parakeet feathers, parrot feathers, finch feathers). With the diagnosis of allergic asthma and rhinitis, inhaled corticosteroid/long-acting beta-agonist combination, intranasal corticosteroid, and leukotriene antagonist therapies were prescribed.

Secondly, eosinophilia work-up and food allergy screening were performed. Aspergillus-specific IgE was negative, and paranasal computer tomography demonstrated mild mucosal thickening at ethmoidal and sphenoidal sinuses. Eosinophilic granulomatosis with polyangiitis (EGPA) and allergic bronchopulmonary aspergillosis (ABPA) was excluded. A standardized food panel of 12 allergens and a prick-to-prick test with cornbread were negative. Total IgE was detected as 207 ku/L, and the basal tryptase level was 3.18 ug/L. 

Finally, an oral cornbread challenge test was performed. Two hours after consuming cornbread, he experienced flushing, sweating, conjunctival erythema, and dyspnea with bilateral rhonchi. Anaphylaxis treatment was administered immediately. Since the patient could consume corn alone, the additives in the bread were examined. Guar gum-specific IgE, which is used as a thickening agent, especially in gluten-free bakery products, was high (49.2 kuA/L, class 4), whereas tryptase level was found very close to basal level (4.47 ug/L).

## Discussion

Three case reports were found when the keywords "guar gum" and "anaphylaxis" were searched in PubMed [6-8]. The first case was a patient who developed anaphylaxis minutes after consuming soy drink and pasta (Tortelline®) in 2002. Although IgE specific for guar gum was negative in this case, the basophil activation test was positive [6]. There was an allergic reaction during a dental procedure in the second case written in 2005. Guar gum was used as a preservative agent in the lidocaine gel, and the patient did not react to the lidocaine challenge. Prick tests with gum derivatives revealed positive results but no specific IgE. Tryptase was also examined in this case, but no rise was noted [7]. In the third case, which was reported in 2007, a 52-year-old patient consumed slimming food (Gerlinea [France]) and ASA together, so aspirin was accepted as a factor that increased allergic side effects. Both gum prick tests and specific IgE (27.2 kuA/L) were positive in this case [8].

Tryptase was tested in only one case in the literature [7], and it was not elevated. Unlike them, we found a very high tryptase in case one. In addition, case one is a very rare example of anaphylaxis. Protracted anaphylaxis is defined as a condition that met the anaphylaxis criteria that lasted at least four hours [9]. A patient described in the literature developed urticaria accompanied by nausea, vomiting, and cramp-like abdomen pain the following day. Interestingly, the tryptase level remained elevated in the blood sample on the third day [10]. Similarly, in case one, other symptoms of anaphylaxis appeared 15 hours after urticaria and tryptase levels were found to be positive after 18 hours. In addition, symptoms persisted for four days despite treatment. Therefore, it was considered protracted anaphylaxis, which is an unusual form of anaphylaxis.

While specific IgE positivity had been determined in only one patient so far [8], we demonstrated the highest serum-specific IgE values in the literature in both patients. Although positive predictive values for common food allergies have been established, no study has shown a predictive value for guar gum [11]. However, values greater than 17.5 kuA/L could strongly be assumed to indicate an IgE-mediated guar gum allergy.

In the case published in 2007, it was demonstrated that concomitant use of guar gum and ASA exacerbated anaphylaxis [8]. There were no facilitating factors such as taking drugs or exercising to enhance the reaction in our cases.

Patients' allergy histories were not recorded in the published cases, or they had no known allergies [6-8]. Case two was allergic to pollen and bird feathers and was diagnosed with allergic asthma and rhinitis. In this regard, it is possible to hypothesize that having several atopic diseases may predispose one to guar gum hypersensitivity.

Allergies to food additives should be considered in cases when patients have allergic symptoms to several different foods and/or packaged forms of foods that are well tolerated when prepared at home [12].

## Conclusions

As far as we know, case one is the unique form of prolonged anaphylaxis and guar-gum hypersensitivity in which tryptase increased 10-fold with level 6 guar gum-specific IgE positivity. We were able to demonstrate the IgE-mediated immune mechanism in both cases. We instructed our patients to read food labels and advised them to avoid guar gum and use adrenaline auto-injectors when necessary. No reaction was detected in the six-month follow-up of both patients.
